# Spectro-Microscopy of Individual Pt–Rh Core–Shell Nanoparticles during Competing Oxidation and Alloying

**DOI:** 10.1021/acsnano.5c07668

**Published:** 2025-07-30

**Authors:** Jagrati Dwivedi, Lydia J. Bachmann, Arno Jeromin, Satishkumar Kulkarni, Heshmat Noei, Liviu C. Tănase, Aarti Tiwari, Lucas de Souza Caldas, Thomas Schmidt, Beatriz Roldan Cuenya, Andreas Stierle, Thomas F. Keller

**Affiliations:** † Centre for X-ray and Nano Science (CXNS), 28332 Deutsches Elektronen-Synchrotron (DESY), Hamburg 22603, Germany; ‡ Department of Interface Science, 28259 Fritz-Haber-Institut der Max-Planck Gesellschaft, Berlin 14195, Germany; § Department of Physics, University of Hamburg, Hamburg 22607, Germany

**Keywords:** in situ spectro-microscopy, Pt–Rh core–shell nanoparticles, facet-dependent oxidation–reduction, correlative approach, XPEEM, SEM−EBSD, AFM

## Abstract

The surface chemical composition of supported single Pt–Rh core–shell nanoparticles was studied to understand the Rh behavior in oxidizing and reducing gas environments using spectro-microscopy with high spatial resolution. We combined *in situ* X-ray photoemission electron microscopy with *ex situ* scanning electron-, atomic force-, and scanning Auger-microscopy to distinguish Rh oxidation–reduction, dewetting–sintering, and alloying–segregation during the course of the experiment. A more than 20% higher Rh 3d_5/2_ oxide to metal photoemission intensity ratio for the Rh layer on top of the Pt-core was found as compared to the bare strontium titanate (STO) oxide catalyst support in close vicinity, where Rh/RhO_*x*_ nanoparticles are forming. At elevated temperatures, Rh diffuses into the Pt particle, and this alloying at the Pt metal surface competes with Rh oxidation, whereas the Rh/RhO_*x*_ nanoparticles on the STO support are observed to sinter under identical oxidizing and temperature environments. A nanoparticle facet-dependent analysis of selected Pt-core nanoparticles suggests that Rh oxidation is most advanced on a small nanoparticle with a low coordination top facet that we indexed by electron backscatter diffraction, demonstrating the strength of our correlative approach.

## Graphical abstract

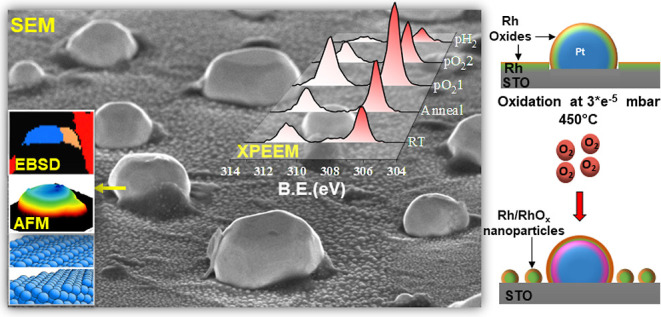

## Introduction

Core–shell nanoparticles with controlled geometry, structural arrangement, and alloy composition are in use today in diverse applications such as the biomedical field, for sensors and electronic and catalytic applications.^1^ Nanoscale catalysts comprise a diverse range of metals and play a pivotal role in enabling efficient chemical transformations essential for sustainable energy production, pollution mitigation, and industrial synthesis.^2–6^

Platinum (Pt) as one of the most prominent metal catalysts is utilized in heterogeneous gas-phase catalysis due to its exceptional catalytic properties, including high surface energy, strong adsorption capabilities, and resistance to oxidation under reaction conditions. Pt efficiently activates and dissociates reactant molecules, such as O_2_ and CO, which is crucial for reactions like the oxygen reduction reaction.^7–9^ Alloying Pt with the highly active Rhodium (Rh) in heterogeneous catalysis enhances electron transfer during catalytic steps, such as the O_2_ reduction or CO oxidation. This combination optimizes adsorption energies for reactants and intermediates,^10–13^ improving thermal stability, catalytic activity, and selectivity. The catalytic behavior of core–shell nanoparticles is still largely unexplored despite successful demonstrations of core–shell catalysts in electrocatalysis,^14^ where changes in electronic properties^15^ or the strain state^16,17^ of the shell atoms induced by the underlying core resulted in enhanced catalytic performances. The shell can also protect the core from corrosion or dissolution.^18^

Oxidation and reduction environments represent two fundamental and opposing regimes to which heterogeneous catalyst nanoparticles are often exposed during their catalytic cycles.^19–24^ The catalytic activity of Pt nanoparticles is highly dependent on the nanoparticle size and shape comprising distinct surface facets with specific atomic arrangements and electronic properties.^23,25–28^ Nanoparticles with high-index facets often exhibit enhanced catalytic performance due to their higher density of low-coordination atoms that serve as active sites. In particular, surface facet-specific features like steps or kinks play a crucial role in the catalytic performance of metal nanoparticle-based catalysts.^29–31^ Metal oxides can act as active sites or passivating layers, directly influencing the catalytic activity of the nanoparticles.^31–33^ During the catalytic reaction, the elevated temperature and reactive oxygen species promote segregation and diffusion. Conversely, H_2_-rich reduction environments may induce a reversion of surface oxides to their metallic state, the desorption of the adsorbed species, and at least partial desegregation and realloying.^34,35^ However, to study such catalyst nanoparticles in dynamic environments requires sophisticated *in situ* techniques. A microscopic view on the chemical composition and morphological changes with a nanoscale spatial resolution can increase the insight beyond the understanding from particle ensemble averaging experiments. Transmission electron microscopy has become a cornerstone technique in recent years for studying chemical processes under ambient pressure and high temperature environments, enabling atomic-scale observation of structural dynamics and surface chemistry.^20,24,36–38^ As one example, for Pt–Rh nanoparticles in an initial core–shell arrangement, a thermodynamically driven elemental mixing was observed during annealing in vacuum.^39^ Furthermore, the compositional distribution of a single Pt–Rh alloy nanoparticle under oxidizing–reducing conditions was investigated by Bragg coherent X-ray diffraction imaging. Information on the Rh segregation under oxygen atmosphere outward toward a core–shell arrangement was deduced from compositional strain profiles obtained from the 3D lattice strain, providing only indirect evidence on the compositional distribution.^34^ Till now, a direct spectroscopic evidence of the elemental near-surface compositions and oxidation states of supported single Pt–Rh nanoparticles in catalytically relevant oxidation and reduction environments is missing. The emergence of spectro-microscopy offers unprecedented opportunities to analyze the behavior of nanoparticles at the atomic and nanoscale level, enhancing our understanding of their role in catalytic processes.^40^

Here, we link X-ray photoemission electron microscopy (XPEEM) combined with low-energy electron microscopy (LEEM) via a correlative approach to scanning electron (SEM), atomic force (AFM), and scanning Auger (SAM) microscopy to investigate the surface chemistry and morphology of Pt–Rh nanoparticles in oxidation and reduction environments. We employ epitaxially grown Pt–Rh core–shell nanoparticles supported by a (100)-oriented strontium titanate (STO) single crystal. In this report, the Pt–Rh core–shell nanoparticles serve as a model system providing a well-defined structural arrangement that enables controlled studies of metal atom diffusion and segregation on the particle, in comparison to the situation on the oxide support.^29^ By comparing the behavior of Rh on these two surfaces using XPEEM, we gain insights into how the supported metal catalyst,^30,41,42^ i.e., the Pt nanoparticles, and the metal oxide carrier or support,^43^ i.e., STO reference, affect the surface characteristics of the Rh under oxidation and reduction conditions. Correlating AFM, SEM, XPEEM, and electron backscatter diffraction (EBSD) permits a crystallographic facet identification of the Pt nanoparticles and reveals that higher-index facets featuring narrow (111) terraces separated by multiple (110) steps and kinks can significantly enhance the Rh oxidation. Overall, this study shows how today’s advances in high-resolution spectro-microscopy can be combined to discern not only substrate carrier specific behavior in oxidation–reduction, dewetting–sintering, and alloying–segregation but also to identify and index nanoparticle facets with enhanced or inhibited oxidation.

## Results and Discussion

### Oxidation–Reduction of Pt–Rh Core–Shell Nanoparticles and of Rh on a Metal Oxide STO Support

Pt–Rh core–shell nanoparticles were synthesized on a niobium-doped STO(100) substrate via dewetting a homogeneous Pt film in a tube furnace in air followed by the overgrowth of a 3 nm-thick layer of Rh under ultrahigh vacuum conditions (see the Experimental Section for details). Both elements have face-centered crystal symmetry with a deviation of 0.1 nm in the lattice constant at room temperature (RT).^44–46^ A SEM image of typical Pt–Rh core–shell nanoparticles is shown in Figure 1a. SEM provides a large field of view (FOV) and allows for statistical analysis to determine the nanoparticle size distribution and the spatial arrangement. Pt particles of sizes ranging from 50 to 1000 nm are evenly distributed across the substrate. To relocate preselected regions of interest (ROIs, see the inset in Figure 1a) in subsequent experiments, we employed ion beam and electron beam-induced deposition (IBID and EBID) in a dual beam FIB–SEM instrument to write Pt-based markers (dotted region in Figure 1a) utilizing a Pt-containing precursor gas.^47^ The hierarchically arranged Pt markers serve as a guide to relocate the ROI during the applied *ex situ* and *in situ* spectro-microscopic experiments and permit a one-to-one correlation on a single nanoparticle level. In this way, the topography of the nanoparticle ensemble in the ROI was investigated by using AFM in tapping mode in air. A high-resolution AFM topographic image of the preselected ROI is shown in Figure 1b. The heights of the Pt–Rh core–shell nanoparticles vary significantly from 50 to 500 nm. Figure 1d represents the height profile of a particle labeled as particle 1 in Figure 1b, with a height of 345 nm.

**Figure 1:**
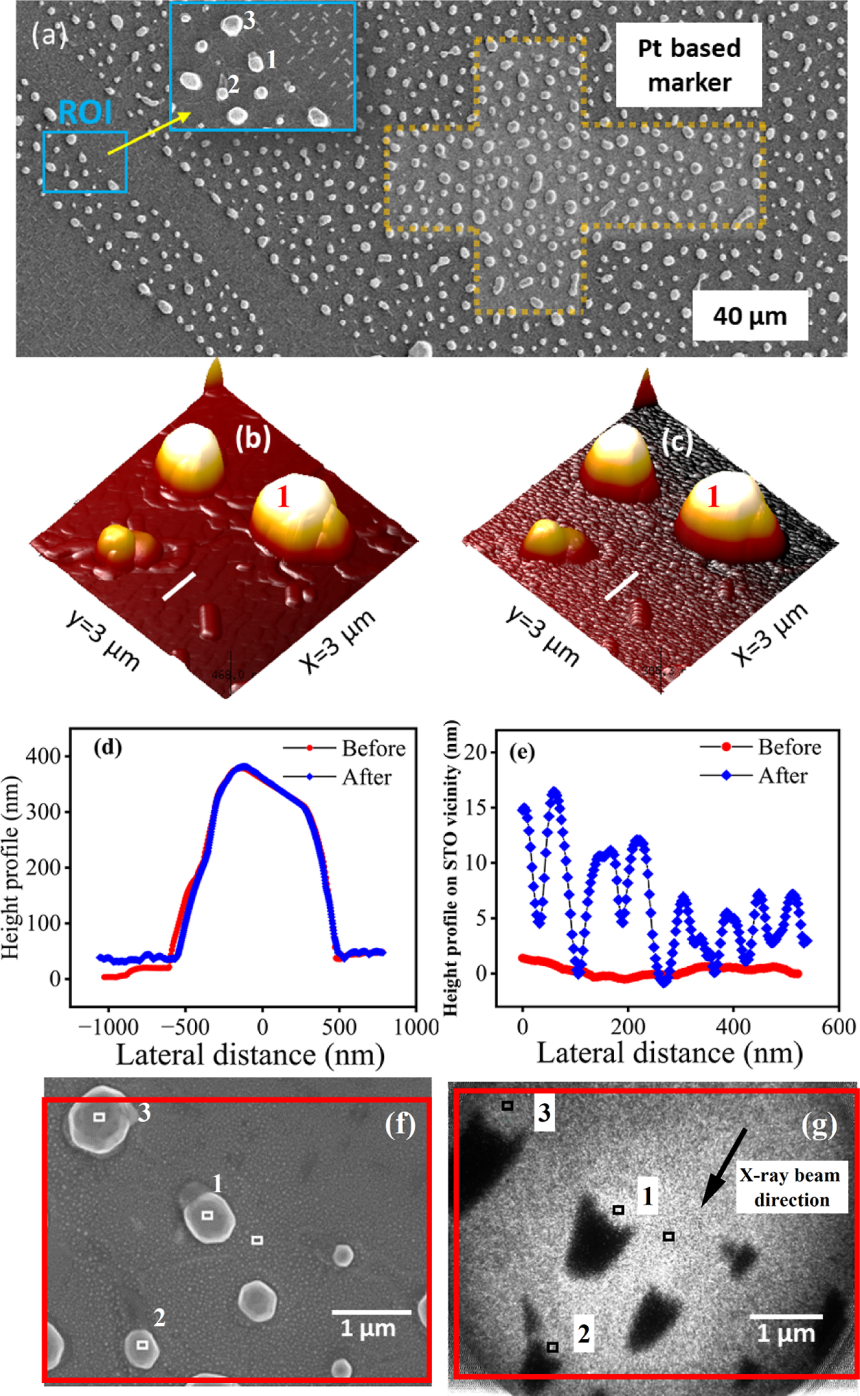
(a) SEM micrograph of the Pt–Rh core–shell nanoparticles’ arrangement on the STO substrate. The small square in the SEM image represents the ROI for in-depth analysis, which was relocated by the Pt-based marker (dotted line). The inset shows a larger view of the selected ROI. (b,c) Comparison of the AFM topography of the ROI before and after the *in situ* experiment. (d) AFM height profiles of particle 1, labeled in Figure 1b,c, and (e) the Rh height distribution in the vicinity on the STO before and after the *in situ* experiment. (f) SEM image after the *in situ* experiment and (g) Rh XPEEM image at a photon energy of 390 eV of the corresponding ROI at RT. The arrow shows the direction of the X-ray beam and the red frames indicate the registered common image region.

#### *In Situ* XPEEM Spectro-Microscopy

First, we discuss and distinguish the behavior of Rh in the shell of Pt–Rh core–shell nanoparticles and Rh on the STO in its direct vicinity. The surface characteristics of the STO supported Pt–Rh core–shell nanoparticles and the nearby Rh were systematically investigated by collecting microscopic XPEEM image series after each sequential treatment, including annealing, oxidation, and reduction. The consecutive treatments are summarized in Table 1. The XPEEM experiment was conducted quasistatically by stepwise adjusting the system conditions, i.e., temperature, pressure, and gas environment, followed by cooling to RT after each treatment. After allowing for equilibration, the XPEEM data were collected to follow the Rh behavior on the sample surface. XP spectra are extracted from the XPEEM image series as outlined below permitting us to determine elemental composition ratios and oxidation states from single nanoparticles with high spatial resolution.

**Table 1:** Temperature and Gas Environments Applied in between the Acquisition of Subsequent XPEEM Maps

abbreviations	treatment	temperature (°C)	pressure (mbar)
RT	room temperature	RT	3 × 10^–10^
anneal	annealing	350	7 × 10^–9^
pO_2_1	first oxidation (O_2_)	350	5 × 10^–6^
pO_2_2	second oxidation (O_2_)	450	3 × 10^–5^
pH_2_	reduction (H_2_)	450	5 × 10^–6^

We employed the LEEM/XPEEM microscope SMART at the UE49PGM undulator beamline of the BESSY II synchrotron light source at the Helmholtz Center Berlin (HZB)^48,49^ (see the Experimental Section for details). To ensure complete observation of the processes, we carefully chose a FOV spanning 6.14 μm in LEEM mode. The bright field LEEM mode was utilized to monitor the ROI throughout the whole *in situ* experiment. This mode provided useful structural contrast but did not provide direct chemical information (Figure S1 in the Supporting Information for LEEM images). The complete chemical information on the surface was obtained by XPEEM. Figure 1g represents the Rh XPEEM image at a photon energy of 390 eV at the initial condition with an FOV of 6.14 μm. The arrow in Figure 1g indicates the direction of the X-ray beam. The dark features in Figure 1g arise from shadowing induced by the small angle of incidence of 21° of the X-rays and the large height of the particles. The sample was exposed to air during transfer for *in situ* measurements.

Prior to measurement, the sample underwent H_2_ cleaning at 250 °C for 60 min to reduce the carbon contamination. Figure S2 in the Supporting Information presents the C 1s XP spectra of the sample before and after the H_2_ cleaning. The XP spectra clearly show that the amount of carbon is reduced but the contamination persists even after the cleaning treatment. To gain surface chemical information, we recorded a series of XPEEM images on Pt 4f and Rh 3d orbitals within its corresponding energy window. The images were taken at photon energies of 150 eV for Pt and 390 eV for Rh at each experimental condition. The information depth of Pt and Rh at these photon energies were calculated to be 1.28 and 1.22 nm, respectively, elucidating the XPEEM’s high surface sensitivity at these photon energies (see the Supporting Information).^50^ For a comprehensive analysis of individual nanoparticles, we collected for each condition and each element, e.g., Pt, Rh, and O, an image series, where each image is taken at a discrete energy value, and the whole XPS peak is obtained from the stack of images from all pixels or selected ROI’s. We systematically extracted spectra from Pt 4f and Rh 3d XPEEM images (see the Supporting Information for the complete methodology of data extraction). Here, we arbitrarily choose particle 1 to discuss the role of the two different surfaces on the Rh behavior, the surface on the Pt particle, and the bare STO. Two 80 × 80 nm^2^ ROIs (see the squares in Figure 1g) were selected: one on particle 1 and the other nearby on the STO for a comparative analysis of the Rh behavior under an oxidizing and reducing environment. Figure 2a–c represents the evolving spectra extracted from the XPEEM data at the five applied conditions (see, e.g., Table 1), from the Pt 4f XPEEM images on the particle region and the Rh 3d XPEEM images on both the particle and the STO region. No Pt was detected on the STO outside the Pt particle region, as all Pt was concentrated within the Pt nanoparticles due to dewetting and is therefore omitted here.

**Figure 2:**
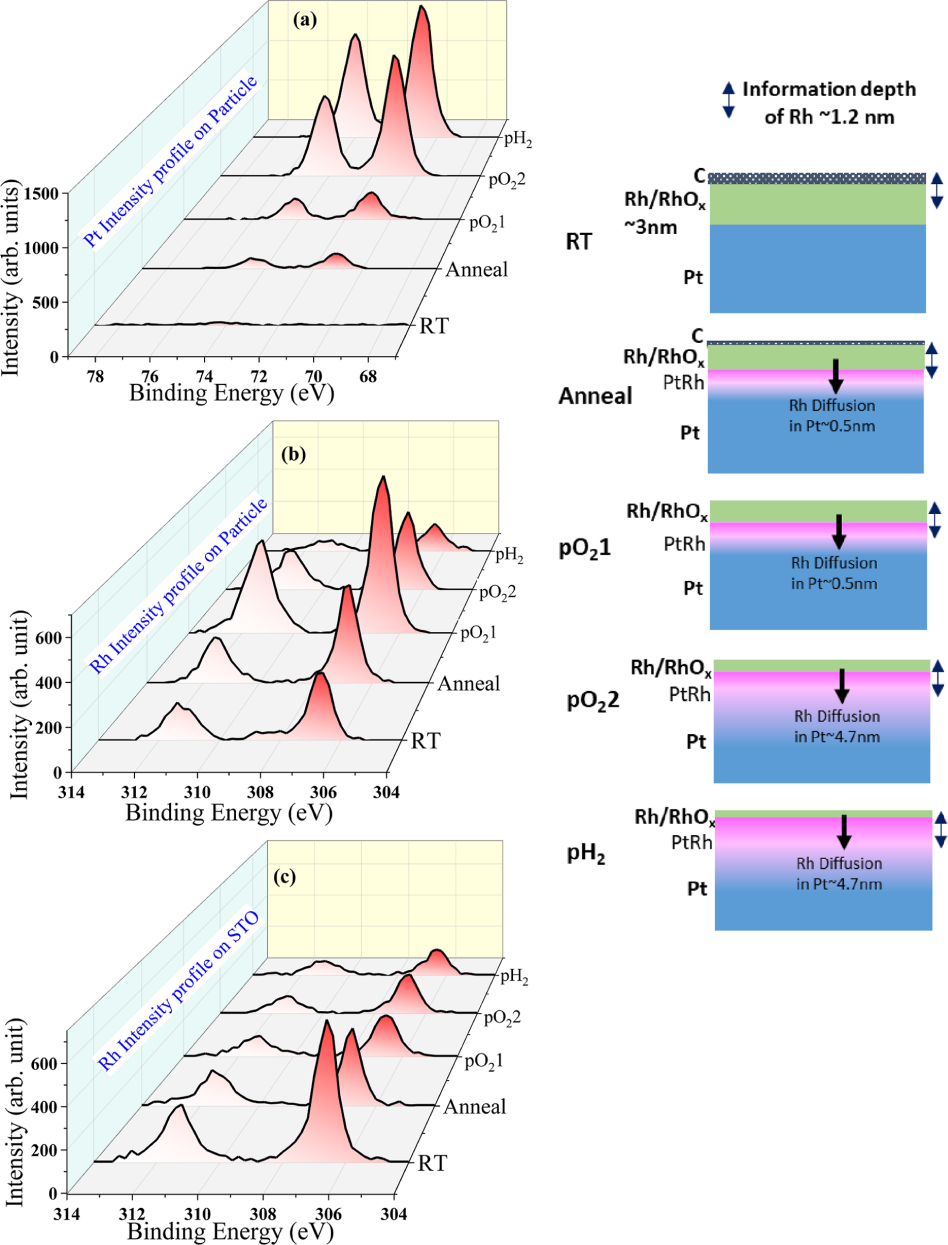
Intensity distribution of (a) Pt 4f spectra on top of the Pt particle, (b) Rh 3d spectra on top of the Pt particle, and (c) in the vicinity on STO extracted from the XPEEM images at each experimental condition. The sketch on the right side is a schematic representation of the Rh behavior on the Pt particle at each experimental condition.

In the initial condition (RT), at RT and a base pressure of 3 × 10^–10^ mbar, the presence of the metallic Rh doublet peaks 3d_5/2_ and 3d_3/2_ at binding energies of 306.72 and 311.30 eV, respectively, arising from the spin–orbital splitting, indicates a homogeneous Rh overgrowth in the selected ROI. Also, the doublet peak positions are in good agreement with literature.^51^ The absence of any Pt 4f signal in the spectra in Figure 2a at RT on the Pt particle is a direct result of the core–shell arrangement of the nanoparticle. Since 3 nm thick Rh covers the top of the Pt particle and the information depth of Pt at 150 eV photon energy is calculated to be 1.28 nm, the detection of Pt is impeded at the initial condition RT. Moreover, the carbon contamination (see Figure S2 in the Supporting Information) is further attenuating the Pt signal.

Figure 3a,b shows the Rh 3d XP spectra on top of the Pt particle and in its close vicinity on the STO support for all environmental conditions applied during the course of the *in situ* experiment. All XP spectra were fitted using CasaXPS,^52^ see the Experimental Section. The fitting parameters such as the binding energy (BE), full width at half-maximum (FWHM), and the calculated intensity ratio of both the Rh oxide and the Rh alloy with respect to the total amount of Rh are summarized in Tables S1 and S2 in the Supporting Information. The fitting results reveal the existence of two different Rh oxide species, Rh^3+^ and Rh^4+^, at binding energies of 307.72 and 308.50 eV that can be identified as two peaks at the left side of the metallic Rh 3d_5/2_ peak in Figure 3a on top of the Pt particle. One of these oxide species at 308.50 eV is identified as the native oxide formed as a result of the overgrowth process and subsequent exposure to ambient conditions.^53^ The intrinsic tendency of Rh to form crystalline oxides, such as Rh_2_O_3_ or rutile-type RhO_2_, contributes to the formation of this native oxide layer.^28,53^ Note that obviously only the Rh^3+^ oxide species is found as Rh_2_O_3_ in the STO region. This variation in oxide species distribution likely originates from a slightly different Rh thickness on top of the 345 nm high Pt particle as compared to the flat STO substrate and/or associated surface roughness variations.

**Figure 3:**
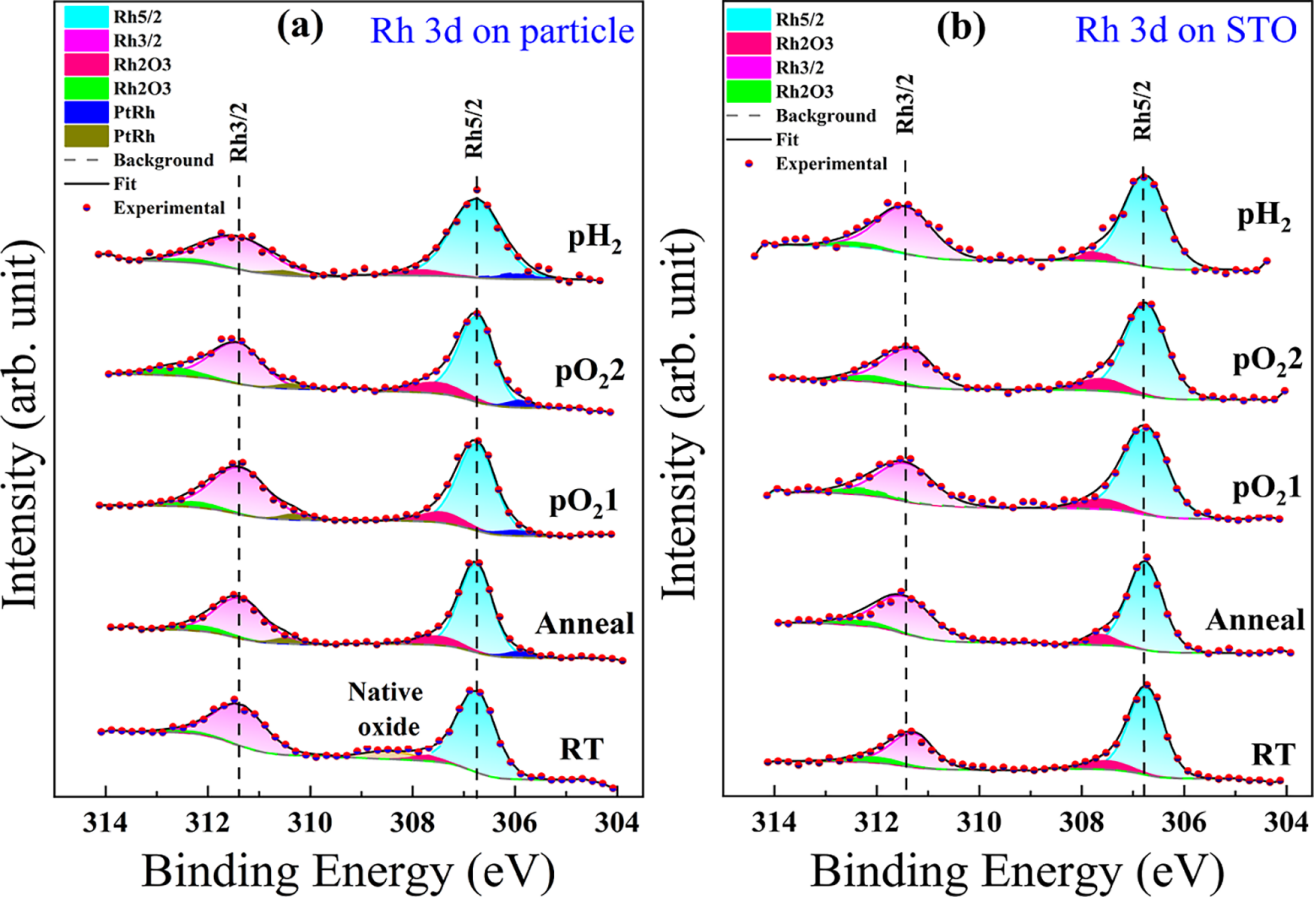
Deconvoluted X-ray photoelectron spectra of (a) Rh 3d on top of the Pt particle and (b) in its vicinity on STO after each experimental condition. The two broad peaks correspond to Rh 3d_5/2_ and Rh 3d_3/2_. Dots are the experimental data points, the black solid line represents the fit result, and the dashed line is the background. Colors indicate the contributions of the metal, different oxide, and alloy species. Fitted parameters are summarized in Tables S1 and S2 in the Supporting Information.

After annealing at 350 °C in UHV for 30 min (anneal), the emergence of doublet peaks of Pt 4f at binding energies of 71.06 and 74.36 eV may be attributed to an improved surface cleanliness as a result of the heat treatment, which successfully reduced the presence of carbon impurities.

The fit results of the Pt 4f signal from the region on top of the Pt particle are shown in Figure S3 in the Supporting Information, indicating a sole metallic state of Pt throughout the course of the experiment. Interestingly, the intensity of the total Rh signal is decreasing on the STO substrate, see, e.g., Figure 2b,c, which may indicate the onset of Rh nanoparticle nucleation and Rh dewetting on the STO substrate from the initially homogeneous thin film. The formed Rh nanoparticles have a height larger than the initial film thickness of 3 nm and correspondingly constitute a smaller surface coverage. In turn, the larger height heterogeneity of the Rh nanoparticles leads to a decreasing total Rh intensity in view of the limited information depth of Rh of 1.22 nm, as discussed above. Surprisingly, the inverse trend is observed in the region on top of the Pt particle. We determine an increase in total Rh intensity by 48.4% as compared to RT. At the same time, the fits of the Rh XP spectra indicate that on the Pt particle, only the Rh^3+^ oxide species persists, which also remains present on the top of the surrounding STO after the annealing, which is explained by the higher stability of Rh_2_O_3_ as compared to the native oxide.^54^ Also note the appearance of two additional peaks at binding energies lower than the Rh 3d_5/2_ and 3d_3/2_ metal peaks, which are solely visible in the top region of the Pt particle. Since Rh is initially located on top of the Pt particle due to the core–shell arrangement, it will diffuse into the Pt matrix due to its excellent miscibility and alloying tendency with Pt, once at an elevated temperature the diffusion is sufficiently high. When electrons are shared between atoms, their electrostatic attraction to the atomic nuclei is reduced. In turn, the electrons are less tightly coupled to the atom, and their BE is lower. For a bimetallic system, the mutual BE is even lower than for a pure metallic element, leading to a peak shift in the XP spectrum toward a lower BE from the pure metal to the alloy.^55,56^ Therefore, we assign these arising peaks to a partial Pt and Rh alloying/intermixing due to the diffusion of Rh atoms in the Pt particle as a result of the heat treatment. We assume the actual temperature to exceed the set value by 50–70 °C due to a nonoptimal thermal contact of the sample and isolation by the STO substrate. Considering this offset, at an estimated temperature of ∼420 °C the diffusion length of Rh in Pt for a holding time of 30 min is calculated to be 0.5 nm.^34,57^

We further determined the oxide (*I*_oxide_/*I*_total_) and alloy intensity ratio (*I*_alloy_/*I*_total_) of Rh with respect to the total amount of Rh (*I*_total_) based on the individually assigned Rh intensities of oxide (*I*_oxide_) and Pt–Rh alloy (*I*_alloy_) obtained from the respective fitted areas in the XP spectra (see Tables S1 and S2 in the Supporting Information). There is approximately 4.1% of alloy after annealing at 350 °C within the top few nanometers sensed by the surface sensitive XPEEM. Figure 4 shows the Rh oxide to metal intensity ratios on the Pt particle compared to the close-by STO along with the intensity ratios of the Pt/Rh alloy on the Pt particle during the course of the *in situ* experiment for all applied experimental conditions.

**Figure 4:**
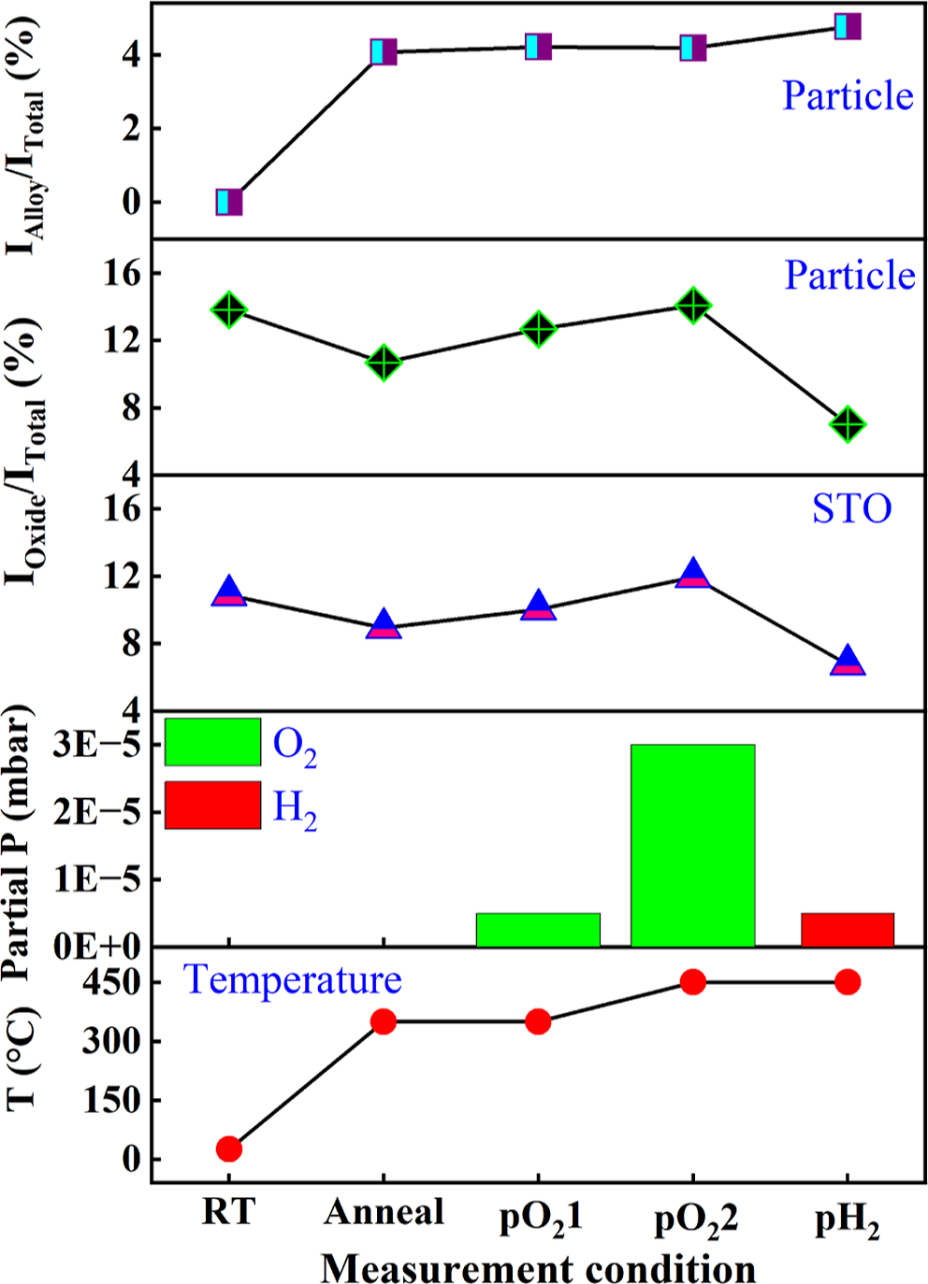
Intensity ratio of Rh oxide and the Pt–Rh alloy as obtained from the Rh 3d_5/2_ peak for the Pt particle and the STO vicinity.

Upon the first oxidation (pO_2_1), at 350 °C under pO_2_ = 5 × 10^–6^ mbar for 30 min, there is a significant increase in the Pt signal on the particle, see e.g., Figure 2a, possibly as a result of the partial alloying of Pt–Rh at the particle interface. At the same time, the Rh intensity is also significantly growing; see e.g., Figure 2b, and the Rh oxide to metal intensity ratio within the Pt particle region is slightly increasing from 10.7 to 12.7%. This phenomenon can be explained by the fact that due to its oxyphilic nature,^58^ Rh is getting more oxidized in the applied oxidizing environment. Since the temperature is the same as that during the annealing process (anneal), the diffusion length of Rh in Pt is expected to be similar. While the increase in total Rh intensity under this condition cannot be solely explained by the competing effects of Rh oxidation and diffusion, it is rather assigned to a complete removal of carbon from the particle surface as a result of the heat treatment under oxygen. Due to the limited information depth of Rh of 1.22 nm, no change in the intensity ratio of the alloy on top of the Pt particle was observed from condition anneal to pO_2_1, as indicated by Figure 4 and Table S1. On the STO substrate, on the other hand, the overall Rh intensity gradually decreased. This reduction indicates the continuous tendency of Rh sintering and particle formation on the STO substrate, which results in less surface coverage. In spite of the increased Rh nanoparticle height accompanying the growth and sintering, the Rh oxide to metal intensity ratio on STO increased from 9.0% to 10.0% (Figure 4 and Table S2).

After further oxidation, at a higher *T* = 450 °C and higher pO_2_ = 3 × 10^–5^ mbar (pO_2_2) for 30 min, the Rh oxide to metal intensity ratio continues to grow for both the Pt particle and the STO region under the more severe oxidation condition, as illustrated in Figure 4, Tables S1 and S2. Note that the information depths of XPEEM in metallic Rh, 1.22 nm, and RhO_*x*_, 1.35 nm are very close.^50^ At the set temperature of 450 °C, estimated to be *T* ∼ 520 °C, the diffusion length of Rh in Pt is ∼4.7 nm, suggesting that in this condition a significant Rh portion of the originally around 3 nm thick Rh layer diffused into Pt and is hidden from the detection by XPEEM, as the diffusion length exceeds the information depth in the Rh/RhO_*x*_ shell. In line with this observation of Rh diffusion into the Pt matrix at a higher temperature, Figures 2, 4 and Table S1 suggest that the Rh diffusion is dominating over Rh oxidation at this condition, which results in a substantial reduction in the total Rh intensity, while the Pt intensity is rising. Interestingly, the intensity ratio of the Pt–Rh alloy remains the same from (pO_2_1) to (pO_2_2), further indicating the limited XPEEM information depth. The slight decrease of the Rh signal on STO indicates that the sintering of the Rh/RhO_*x*_ nanoparticles is not advancing anymore, likely stabilized by Rh oxidation.

Following the exposure to reducing condition at 450 °C and pH_2_ = 5 × 10^–6^ mbar (pH_2_) for 30 min, the reduction of the Rh oxides on both, the top of the Pt particle and in its close vicinity on STO is observed, as shown in Figure 4. Simultaneously, the further increasing and declining total Pt and Rh intensities, respectively, indicate a continuous diffusion of Rh into Pt, in line with an increased Pt–Rh alloy signal rising from 4.1% to 4.8% on the Pt particle (see Figure 4 and Table S1 in the Supporting Information). This behavior demonstrates the strength of the reduction process in which RhO_*x*_ transforms to its more stable metallic form and the Rh realloying toward the Pt particle core is reinforced.^34^

#### Correlative Post Analysis

To independently confirm the observed Rh sintering and nanoparticle formation throughout the whole *in situ* experiment, we performed a series of postexperiments using AFM, SEM, and SAM. Figure 1c illustrates a 3D representation of an AFM topographic image collected after the oxidation–reduction treatment, and Figure 1d shows the height profile of particle 1, from which the XP spectra were retrieved. No noticeable variation in particle height was observed during the *in situ* experiment, indicating the nanoparticle’s stability under these conditions. However, the Rh distribution on the STO surface before and after the *in situ* experiment has changed. In Figure 1e, the height profile on the STO region collected across the white line in Figure 1b,c is plotted. Here, a clear transformation is observed, indicating Rh particles ranging from 5 to 10 nm in height as a result of the system being subjected to heat in the oxidizing and reducing environments. The SEM image acquired after the treatment illustrated in Figure 1f confirms these findings by showing the dewetting of the Rh film into Rh nanoparticles on the STO surface and in turn a decreased surface coverage. In parallel, we employed correlative SAM, comparing data collected before and after the *in situ* experiment. Figure S4 in the Supporting Information displays the Rh Auger map, both before and after the *in situ* experiment, along with the Auger survey spectra from the STO support. The Rh Auger map obtained before the *in situ* experiment exhibits a higher intensity in the Pt particle region, which significantly decreases after the *in situ* experiment, clearly indicating a net diffusion of Rh into the Pt core. Moreover, the Auger survey data revealed an increase in the substrate elements, such as Sr and Ti and reduction in the Rh Auger signal, providing additional confirmation of Rh sintering due to the surface processes occurring in the system.

In summary, the postexperimental analyses by AFM, SEM, and SAM are in excellent agreement with our XPEEM findings and highlight the complex kinetics of surface processes on the Pt nanoparticles and the STO support under oxidation–reduction conditions. Our data indicate that several competing and/or cooperative mechanisms are active during the course of the *in situ* experiment including (i) nucleation and sintering of Rh nanoparticles on the STO support, (ii) Rh oxide formation and reduction, and (iii) alloying–dealloying of Rh that segregates into Pt. Moreover, each of these mechanisms follows its own specific temperature- and gas environment-dependence. Although Figure 4 illustrates an overall similar tendency for the Rh oxide to metal intensity ratio on the Pt particle and the STO, this experiment indicates an overall more than 20% larger Rh oxide to metal intensity ratio on the Pt particle compared to STO. The H_2_ treatment reduces the Rh oxide at least partially to the metal state in both regions. In addition, the AFM results illustrate the largely unaltered height of the Pt nanoparticles, underlining the stability of their structural integrity during the *in situ* experiment. Moreover, the postanalysis confirms that the Rh behavior is distinct in terms of the carrier surface, suggesting no sintering on the Pt surface compared to the bare STO. Figure 5 schematically sketches conclusively the observed behavior of the Rh on the Pt–Rh core–shell nanoparticles and the STO support under the applied oxidation and reduction conditions.

**Figure 5:**
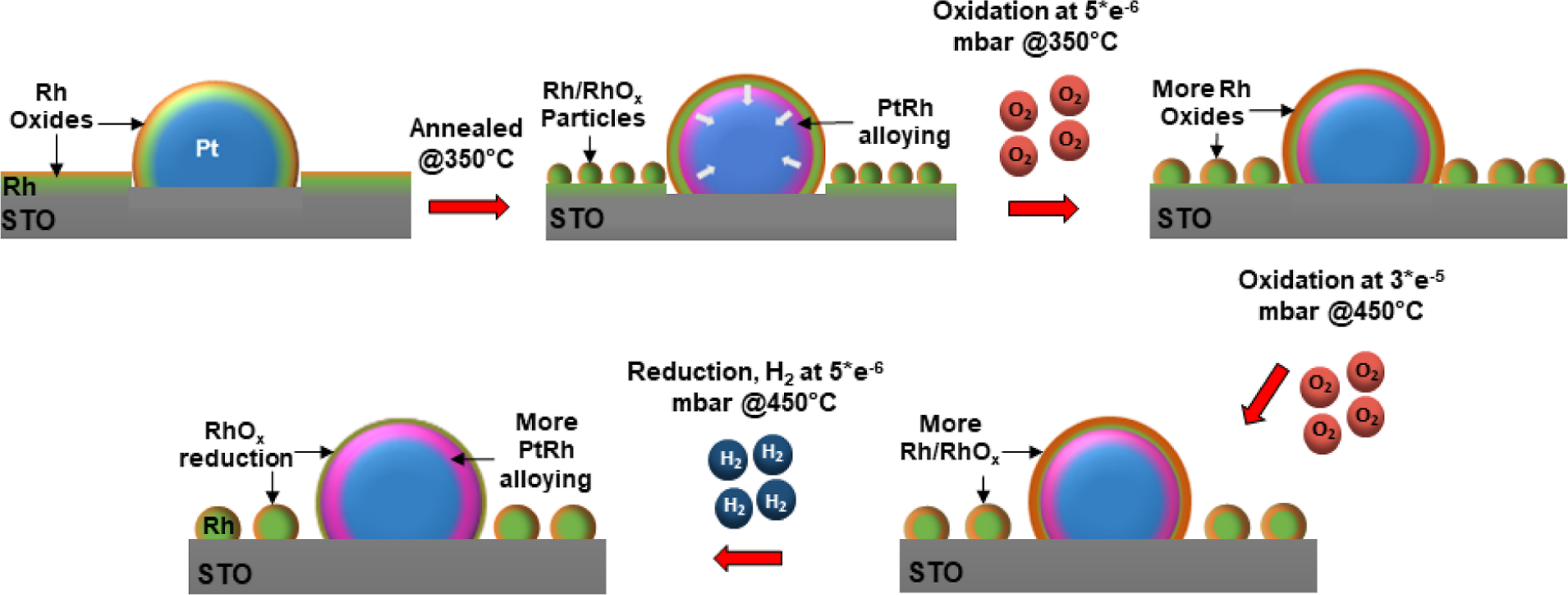
Schematic representation of Pt–Rh core–shell nanoparticles and the Rh on the STO support under the applied oxidation and reduction conditions at elevated temperatures.

### Particle and Facet-Specific Oxidation Behavior

So far, we compared the behavior of Rh in the Pt–Rh core–shell arrangement to that of the bare STO carrier substrate during oxidation and reduction. We now analyze ROIs on selected Pt particles and facets, as in general, the oxide formation can depend on the atomic arrangement of specific nanoparticle’s facets.^19,59^ Rizo et al. state that the oxygen atoms preferentially adsorb on the stepped atoms, facilitating the exchange between oxygen and metallic atoms that enhance the activity.^60^ High-index facets are typically characterized by steps, terraces, and kinks and therefore have a higher surface energy compared to low-coordinated surfaces. We therefore arbitrarily chose three Pt–Rh core–shell nanoparticles of different shapes and surface facets in order to investigate variations in Rh oxidation within an oxidizing environment.

All particles with a height range of 200–500 nm labeled 1–3 are seen in the image-registered SEM and Rh XPEEM images in Figure 1f,g. The lateral size and height of all three particles are summarized in Table S3 in the Supporting Information. To analyze the Rh oxidation behavior on all three particles, the Rh 3d XPEEM spectra at RT and pO_2_1 were extracted from an 80 × 80 nm^2^ ROI centered on top of each of the Pt particles, see, e.g., Figure 1g using the same methodology as described in the first section. The fitted results of the Rh 3d_5/2_ peak at RT and pO_2_1 are shown for all three particles in Figure S5 in the Supporting Information.

At RT, the fits indicate that initially two different oxide species, i.e., Rh^3+^ and Rh^4+^, at binding energies of 307.73 and 308.50 eV for the Rh 3d_5/2_ peak, respectively, are present for all three particles, and no signal of Rh alloying in Pt. Following oxidation at pO_2_1, only Rh^3+^ as a single Rh oxide remains present. We further observe a partial Pt and Rh alloying at this oxidation condition by the existence of a small peak at a lower BE of the Rh 3d_5/2_ peak at 305.93 eV. The existence of two distinct oxides at RT, the stability of Rh^3+^ oxides after oxidation, as well as the alloying of Rh in Pt at pO_2_1 are in-line with the results discussed in the previous section. However, the alloy is present only on particles 1 and 2 at pO_2_1. The intensity ratios of Rh oxide and alloy on all three particles are summarized in Table 2, indicating the highest Rh oxide to metal intensity ratio for both conditions RT and pO_2_1 on the smallest particle 2 as compared to particles 1 and 3. For all three analyzed particles, the Rh oxide to metal intensity ratio increased from RT to pO_2_1.

**Table 2:** Fitting Parameters of Rh on Top of the Three Pt Particles along with the Obtained Facet Indices. The Intensity Ratios Are Given in Percent (%)

particle #	*I*_oxide_/*I*_total_ at RT	*I*_oxide_/*I*_total_ at pO_2_1	*I*_alloy_/*I*_total_ at pO_2_1	identified facets
particle 1 (middle)	13.83	12.67	4.21	(331), (102)
particle 2 (small)	16.19	14.20	4.16	(353)
particle 3 (big)	9.12	11.54		(131), (−104), (7–75)

The Pt–Rh core–shell nanoparticle’s morphology becomes evident from the SEM micrograph images taken with a sample tilt of 70° and the AFM image in Figure 6a,b that were recorded after the oxidation experiment to get a 3D representation. These images show that the particles have distinct surface facets and that the top surfaces of all three particles are tilted.

**Figure 6:**
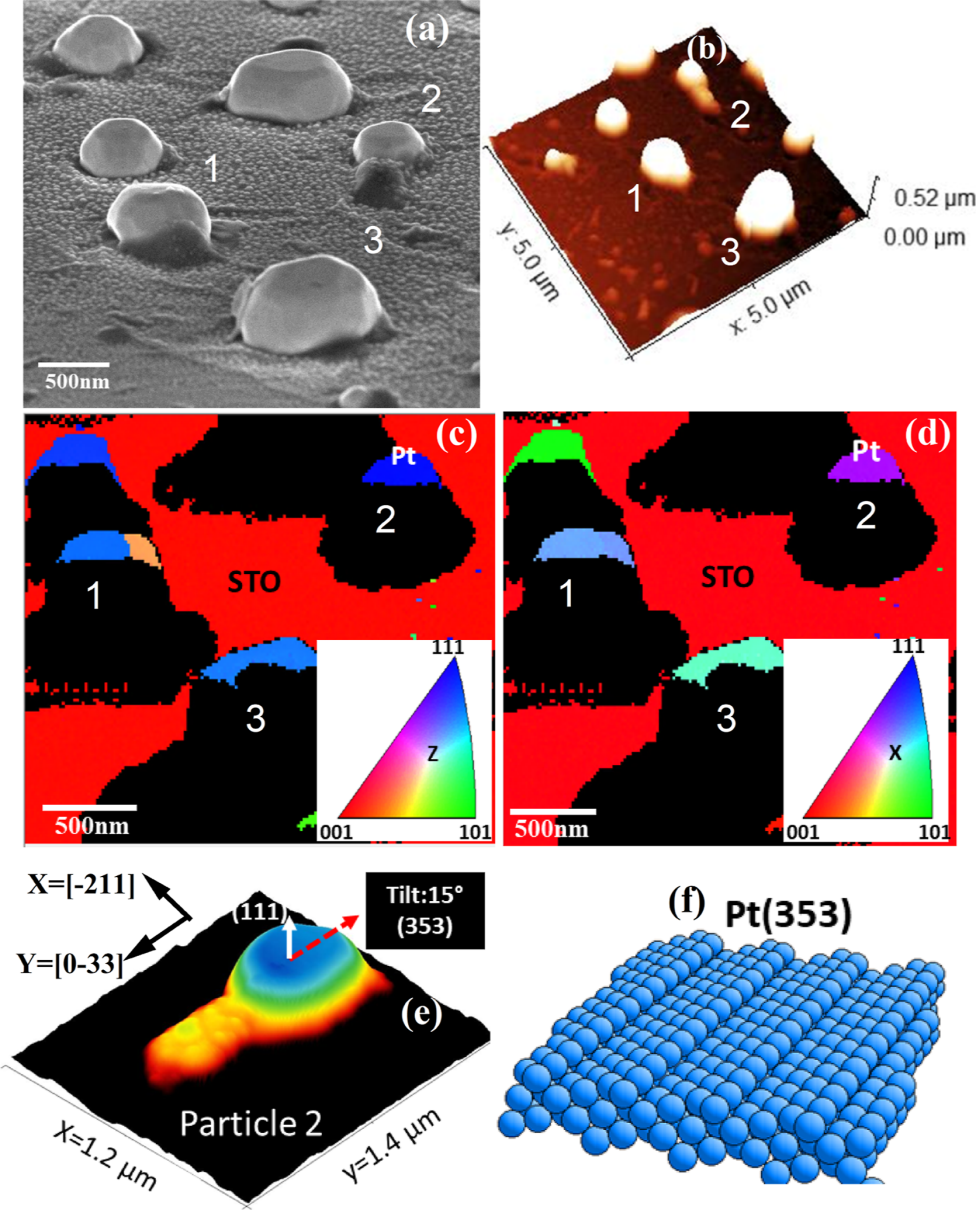
(a) 70° tilted view SEM micrograph, (b) three-dimensional representation of the registered AFM topographic image, and (c,d) two-dimensional out-of-plane (*Z*) and in-plane (*X*) EBSD IPF maps containing the preselected ROI after oxidation. The colors indicate the bulk crystal orientation of the Pt particle or STO substrate as given in the inset. (e) AFM topography of particle 2 in 3D view. The tilt angle is given with respect to the substrate normal corresponding to the nanoparticle’s out-of-plane orientation (white arrow, for particle 2 along (111)), from which the top surface facet orientation (353) is deduced. The black arrows direct along *X* and *Y* and are labeled with the corresponding in-plane crystal orientation. (f) Atomic arrangement of the (353) surface facet.

In order to index these surface facets, we deduce the crystallographic orientation of the Pt particles using EBSD. Accordingly, the local crystal orientation of the ROI containing all three analyzed particles with respect to the substrate surface normal (*Z*-direction) is expressed in the inverse pole figure (IPF) map in Figure 6c. The blue color in the map for particle 2 in the upper right reveals that the out-of-plane orientation of the crystal lattice is aligned with the Pt(111) direction along the surface normal, whereas the STO substrate is determined to be (001) oriented. Furthermore, the EBSD provides information on the crystal orientations in the in-plane direction shown in Figure 6d. The in- and out-of-plane orientations of all three particles are summarized in Table S4 in the Supporting Information. The indices of the surface facets of all truncated particles can be determined by correlating the crystallographic in- and out-of-plane information from EBSD with the surface tilts determined by AFM as follows.

We have constructed the crystal orientation using the Pt FCC crystal structure, and the in-plane and out-of-plane orientations deduced from EBSD using the software VESTA.^61^ The middle column of Figure S6 in the Supporting Information shows the atomic arrangement of the reconstructed particles 1–3 with in- and out-of-plane crystal orientations matching the EBSD results. The right column of Figure S6 displays 2D projections of the 3D AFM topography showing a side view of the given top surface facet, and for comparison, the left column of Figure S6 contains the corresponding 2D AFM heights images. To extract the (*hkl*) Miller indices of a top surface facet, we monitored the in- and out-of-plane rotation angles needed to align it to the EBSD reference, i.e. surface normal along *Z*, and in-plane directions parallel to *X* and *Y*, respectively, see, e.g., Figure S6. The integer Miller indices were obtained from the plane that most closely matches these rotations. Accordingly, Tables 2 and S4 contain the deduced crystallographic indices of the facets of interest. Detailed information on how to use VESTA to determine facet orientation from EBSD and AFM data is provided in the Supporting Information. Figure 6e,f shows an example of the crystal structure visualization, where the in-plane and out-of-plane tilt angles of the top surface of the smallest particle 2 were calculated relative to the EBSD *Y*- and *Z*-axis reference orientations using the AFM image (Figure 6e), indicating that the tilted surface corresponds to a (353) facet (Figure 6f and Table 2).

Image registration of EBSD, AFM, and XPEEM, see, Figures 1f,g, 6a,b and S8 and the Experimental Section for a detailed description, shows that the ROIs used for the XPEEM analysis consist of a single top surface on particle 2, a single top surface on particle 1 crossing a crystal twin boundary, and a multifacet top region with three tilted surfaces on particle 3. Consequently, we calculated the Miller indices of a total of 6 vicinal surfaces to be (331) and (102) on particle 1, (353) on particle 2, and (−104), (131), and (7–75) on particle 3, see also Table S4 in the Supporting Information. Figures 6e,f and S7 show AFM height images of all three particles in 3D and illustrate the corresponding atomic arrangements of all identified facets, highlighting their overall high-index nature as sketched by the Balsac software.^62^ Whereas the (111) facet of Pt particles is known to exhibit a close-packed hexagonal arrangement of atoms, forming a densely packed layer,^63^ atomic arrangements on the top facets of our Pt particles are generally more complex. The top facets on the twinned particles 1, (331) and (102) show atomic arrangements, which include multiple steps of (010) planes and narrow (111) terraces with 2 and 3 atoms between neighboring step edges. Similarly, the atomic arrangement of the (353) facet of particle 2 consists of flat terraces terminated at atomic steps. Each terrace corresponds to a (111) plane with 4 atoms, while the steps are aligned along (010) directions. This configuration leads to an increased density of low-coordinate atoms at the step edges. On top of particle 3, e.g., the (7–75) facet exhibits a series of large (111) terraces with 6 atoms separated by (010) steps, while the (−104) facet has a series of large (100) terraces separated by (101) steps. The (131) facet consists of a stepped surface of the (111) plane but with more frequent step edges and kink sites.

The (353) facet surface on the smallest particle 2 has the highest index and could therefore be the origin of the observed highest Rh oxide/metal intensity ratio. In the case of particle 3, the ROI lies largely on a (7–75) surface. This determination is based on the image registration of the SEM image with XPEEM and AFM with a precision of approximately 15 nm. Considering this margin of error, we estimate that approximately 80% of the ROI is located on the (7–75) surface, and therefore the dominating source of the XPEEM signal. This surface has a lower density of steps and a lower Rh oxide to metal intensity ratio than the (353) surface of particle 2, supporting our argument that surfaces with a lower density of coordinatively unsaturated atoms are less reactive. Conversely, for particle 1, the trend is reversed because both the (331) and (102) surfaces have a higher density of steps. However, it is important to note that in gas-phase catalytic reactions like CO conversion, the topmost atomic surface arrangement is decisive for the activity that can be enhanced by low-coordinated atoms. The oxidation process, however, may propagate to subsurface regions and form oxygen species or even fully oxidized small nanoparticles,^20^ which ultimately affects the oxide to metal intensity ratio.

## Conclusion

Our spectro-microscopic XPEEM experiment of Pt–Rh core–shell nanoparticles in oxidative and reducing gas environments provides chemical insights into the Rh behavior at elevated temperatures and varying pressures. The correlative approach, utilizing image-registration of XPEEM, AFM, SEM/EBSD and SAM, permits a unique view on the single nanoparticle level. We conclude that while Rh on the STO oxide support is strongly sintering and dewetting to form nanoparticles at a sufficiently high temperature, there is a continuous alloying of Rh inside the shell of the Pt–Rh core–shell nanoparticles throughout the whole experiment. The oxidizing or reducing gas environments could further enhance or reduce the net flow of Rh into the Pt core. Implications for catalytic activity are evident from the impact of diffusion and sintering on the top surface atomic arrangement. We further observe an around 20% higher Rh oxide to metal XPEEM intensity ratio on Pt nanoparticles than on the STO oxide support, which we attribute to a stronger metal-/metal oxide-support interaction of RhO_*x*_ and Pt in comparison to STO. Moreover, although we found the highest oxide to metal intensity ratio on the nanoparticle with the highest facet index, our nanoparticle facet-resolved experiment suggests that Rh is not generally more susceptible to oxidation if it is located on a Pt nanoparticle with a higher surface facet index. The surface with the lowest facet index did not lead to the lowest level of oxidation for the facets investigated. This observation may be linked to a proceeding oxidation to subsurface Rh layers, a process affected by several facet-specific steps including oxygen adsorption, dissociation, and ion diffusion into Rh. This indicates that oxidation is not only controlled by the topmost atomic surface arrangement that is often decisive for the activity in gas-phase catalytic reactions. For the design of future catalysts, it is certainly worth considering how most active surface facets can be stabilized in view of the dominating mechanism diffusion and oxidation. The insight obtained from our correlative approach paves the way for a deeper understanding of the catalyst behavior at the nanoscale, offering valuable perspectives for tailored applications in catalysis and beyond. This work may further serve as one example for more comprehensive correlative investigations linking chemical and structural information on the nanoscale.

## Experimental Section

In this study, we employed a sequential process to fabricate Pt–Rh core–shell alloy nanoparticles on a 0.7 wt % niobium-doped STO substrate with (100) orientation (miscut <0.1°). A particular advantage over, e.g., sapphire, is the possibility of niobium doping to increase the electrical conductivity, making it more suitable for electro-microscopic studies such as SEM, SAM, and XPEEM by reducing charging effects and improving signal quality.^64^ The substrate was first subjected to a buffered oxide etch solution (BOE, ammonium fluoride buffered hydrofluoric acid (6:1)) treatment, which involved soaking it in ultrapure water for 10 min in an ultrasonic bath, then etching it for 30 s in BOE before being rinsed in high-purity water, and dried in a stream of dry N_2_,^65^ to induce a titania surface termination. Following that, annealing in a tube furnace in air for 60 min at 950 °C was carried out.^66^ Afterward, the STO substrate was introduced into an electron-beam evaporation chamber, and a 50 nm-thick layer of Pt was deposited. Subsequently, the creation of nanoparticles was achieved through a dewetting process in a tube furnace in air. The furnace was maintained at a temperature of 1200 °C (ramp with 1200 K/min) for 60 min, allowing the Pt layer to undergo dewetting and form nanoparticles. To achieve the desired Pt–Rh core–shell structure, a 3 nm-thick layer of Rh was overgrown in a molecular beam epitaxy chamber, at 300 °C under ultra-high vacuum conditions (∼5 × 10^–10^ mbar).

### Characterization Techniques

#### Scanning Electron Microscopy

The SEM images were taken using the FEI Nova Nano SEM 450 instrument at the DESY NanoLab.^47^ The lower resolution overview SEM image of the hierarchical markers of 40 μm scale size (Figure 1a) was taken with an Everhart–Thornley secondary electron (SE) detector at a 5 kV acceleration voltage. The higher resolution image of the nanoparticles in Figure 1f was recorded at an acceleration voltage of 10 kV with a through-lense detector in the SE mode. Another high-resolution SEM image in Figure 6 was taken at an acceleration voltage of 5 kV in a similar mode with a 70° tilt of the sample stage to get a 3D view of the nanoparticles.

#### Atomic Force Microscopy

AFM topographic images were obtained using a Dimension Icon instrument (Bruker Nano) equipped with a Nanoscope Controller V at the DESY NanoLab to study the surface morphology of the Pt–Rh core–shell nanoparticles.^47^ The AFM images were recorded under intermittent (tapping) mode, with a resolution of 1024 × 1024 and 512 × 512 pixels in Figures 1 and 6, respectively, and their corresponding scan rates are 0.498 and 0.1 Hz.

#### Scanning Auger Microscopy

Auger maps for Rh before and after the *in situ* experiment were obtained with 256 × 256 pixels using the energy windows 269–326 eV for Rh with a PHI 710 Scanning Auger Nanoprobe instrument.^47^ The maps were collected from a 2.5 × 2.5 μm^2^ area. Auger survey spectra were recorded in the energy range of 50–2050 eV with a step size of 1 eV before and after the *in situ* XPEEM experiment. The electron acceleration voltage and beam current were 20 kV and 1 nA, respectively. For quantitative analysis, Rh Auger maps were processed and analyzed using CasaXPS.^52^ Spectra from individual pixels were extracted, but the raw spectra exhibited a high signal-to-noise ratio, complicating direct analysis. To resolve this issue, the spectra were processed using a linear least-squares (LLS) fitting method. The applied LLS fitting incorporates a principal component analysis, breaking down the experimental spectrum into a linear combination of dominant components to minimize residual errors. After LLS fitting, background subtraction was performed on each pixel’s spectrum to quantify Rh signals. The quantified data were then compiled into a spatial image representing the elemental distribution across the scanned area. This combination of LLS fitting and background subtraction improved the accuracy of the map, allowing for a clearer interpretation of the elemental distribution.

#### X-ray Photoemission Electron Microscopy and Low Energy Electron Microscopy

The XPEEM/LEEM data were collected using the SMART microscope operated at the UE49PGM undulator beamline at the BESSY II synchrotron, HZB. The aberration-corrected and energy-filtered LEEM–XPEEM system achieves a lateral resolution of 2.6 nm in LEEM mode and 18 nm in XPEEM.^67,68^ The XPEEM spectra were produced by local integration of pixel intensities from specific regions in a series of images recorded by scanning the sample potential between specific values, tuned in such a way as to detect both Rh 3d and Pt 4f signals with the highest surface sensitivity, i.e., kinetic energy of around 70–80 eV. In this way, each image of the series corresponds to a specific energy value of the XP spectra. The photon energy was selected accordingly. The energy filter allows the detection of energy-selected images with a BE contrast. Before extracting the data at each oxidation and reduction condition, we registered the images of each stack for each image series using ImageJ^69^ to correct for potential drifts. After drift correction, we selected ROIs from which the XP spectra were extracted. The XP spectra were fitted using CasaXPS with a Shirley background.^52^ For peak fitting, a mixture of Gaussian and Lorentzian curves was used. During the fitting, the area of the Rh 3d_5/2_ and 3d_3/2_ peaks was kept fixed at a 3:2 ratio, following standard refs 70–72 . All four marked ROIs in the XPEEM image (Figure 1g), size of 80 × 80 nm^2^, correspond to 23 × 23 pixels. The intensities and energies are calibrated to permit a direct comparison.

#### Electron Backscatter Diffraction

We used EBSD combined with SEM to obtain crystal orientation images using automated indexation of the Kikuchi pattern,^73^ which result from multiple electron Bragg-reflections. EBSD provides information about the out-of-plane and in-plane crystal orientations shown in Figure 6c,d, indicating that the STO substrate with a (100) in-plane orientation was aligned with its edges along *X* and *Y* in the SEM during the EBSD analysis. Pt and STO are indexed at the same time due to the identical crystal symmetry and the small difference in their lattice constants.

#### Image Registration

The image stacks of the XPEEM data were registered as described above. To identify the surface regions from which the XPEEM data were analyzed, we utilized image registration to match the lateral positions in the XPEEM, AFM, and SEM images. Image registration is the process of mapping and geometrically aligning two or more images of similar or different modalities. We utilized a Python-based GUI image registration script. This software facilitates the registration of SEM, AFM, and XPEEM images, enabling the alignment, in this case, to a precision of around 15 nm. The registered images of SEM/XPEEM and SEM/AFM are shown in Figure S8 in the Supporting Information.

## Supplementary Material



## Notes

LEEM images at all *in situ* measurement conditions; C 1s spectra before and after cleaning at 250 °C; calculation of X-ray photoemission information depth of Pt and Rh; data extraction method from XPEEM images; Rh XPS fitted parameters on top of the Pt particle and STO support; Pt XPEEM spectra on particle 1 at all *in situ* measurement conditions; Rh Auger map and Auger survey spectra before and after the *in situ* experiment; Rh 3d XPEEM spectra of all three particles at RT and after first oxidation; parameters extracted from SEM and AFM images of all three particles; description of particle reconstruction in VESTA for facet identification along with its representation and listing the identified surface facets on all three particles; and image registration of SEM/XPEEM and SEM/AFM images (PDF)
