# Supplementary material for: Spectro-Microscopy of Individual Pt–Rh Core–Shell Nanoparticles during Competing Oxidation and Alloying
Source: ACS Nano. 2025 Jul 30;19(31):28516–29. doi: 10.1021/acsnano.5c07668 (PMC12356121; doi:10.1021/acsnano.5c07668)
Supplement: Supplementary file 1 [file nn5c07668_si_001.pdf]

Supporting Information: Spectro-Microscopy of Individual Pt-Rh Core-Shell Nanoparticles During Competing Oxidation and Alloying

Jagrati Dwivedi,[*,†] Lydia J. Bachmann,[†,‡] Arno Jeromin,[†] Satishkumar Kulkarni,[†] Heshmat Noei,[†] Liviu C. Tănase,[¶] Aarti Tiwari,[¶] Lucas de Souza Caldas,[¶] Thomas Schmidt,[¶] Beatriz Roldan Cuenya,[¶] Andreas Stierle,[†,‡] and Thomas F. Keller[*,†,‡]

†*Centre for X-ray and Nano Science CXNS, Deutsches Elektronen-Synchrotron DESY, 22603 Hamburg, Germany*

‡*Department of Physics, University of Hamburg, 22607 Hamburg, Germany*

¶*Department of Interface Science, Fritz-Haber-Institut der Max-Planck Gesellschaft, 14195 Berlin, Germany*

E-mail: jagrati.dwivedi@desy.de; thomas.keller@desy.de

Low-Energy Electron Microscopy (LEEM)

Figure S1 shows the low energy electron microscopy images at a kinetic energy of 5 eV with the field of view of 6.14 µm at all analyzed conditions. The LEEM mode was employed to continuously monitor the region of interest throughout the *in situ* experiment. The contrast of LEEM images becomes dark under oxidizing conditions because oxidation changes the

surface composition and electronic structure of the material.

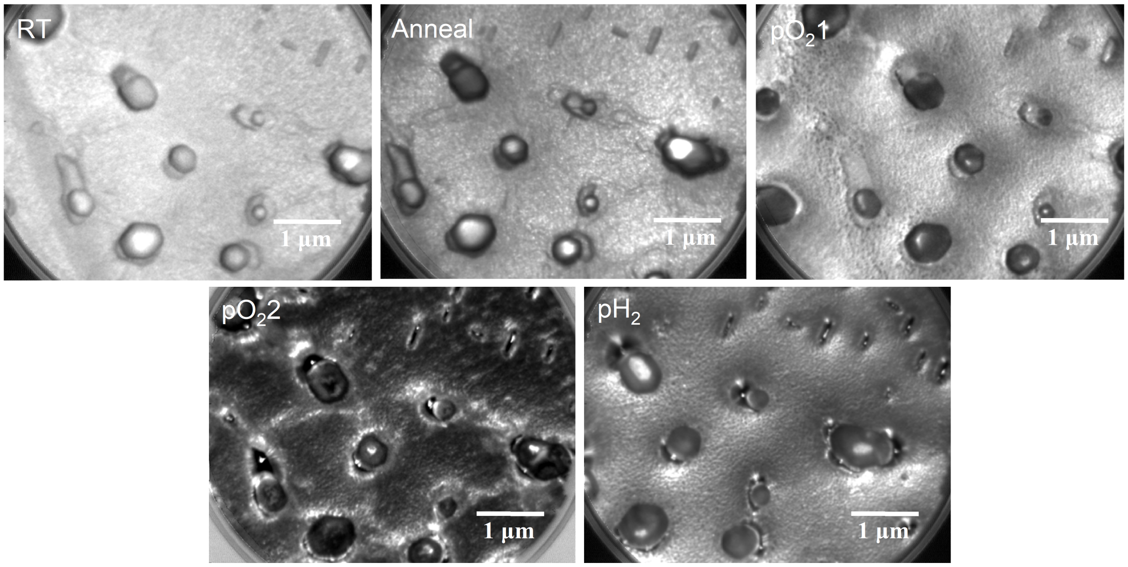

Figure S1: LEEM images at all analyzed conditions. All images were recorded with the same field of view (6.14 µm) at a kinetic energy of 5 eV.

Carbon contamination on sample surface

Figure S2 represents the C1s XP spectra before and after the H_2 cleaning treatment at 250°C for 60 min to reduce the carbon contamination. The XP spectra after cleaning show persistent carbon contamination on the whole sample surface, as evidenced by the C1s peak remaining after cleaning.

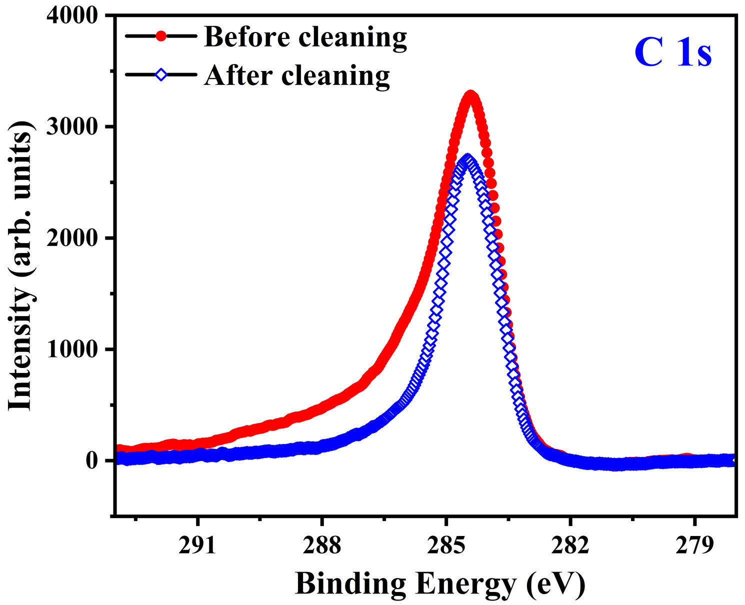

Figure S2: C1s XP spectra before and after the H_2 cleaning at 250°C.

Calculation of information depth

We utilized the NIST electron inelastic-mean-free-path database version 1.2[S1] and QUASES-IMFP-TPP2M[S2] software to obtain the electron inelastic mean free path (IMFP) for Pt at a photon energy of 150 eV and Rh and Rh_2O_3 at a photon energy of 390 eV. We considered the hexagonal corundum structure of Rh_2O_3 with the space group R-3c.[S3,S4] The crystal structure of Pt and Rh are cubic. The resulting IMFP values were determined to be 0.43 nm for Pt, 0.40 nm for Rh, and 0.45 nm for Rh_2O_3. With the XPEEM setup used, only electrons leaving the sample at 90° with respect to the surface are detected, *i.e.*, where the detector is located. The information depth, which represents the depth from which approximately 95% of the total detected electrons originate, is calculated to be 3 x IMFP.[S5] Accordingly, the calculated information depth of Pt, Rh and Rh_2O_3 are 1.28, 1.22 and 1.35 nm, respectively.

Data extraction from XPEEM images

To extract the XPEEM spectra of Pt and Rh from their series of XPEEM images, we utilized the IDL environment-based SMART analysis program. Before importing the XPEEM images into the software, we applied a flatfield correction to subtract the background.

For drift correction, we used the ImageJ software.[S6] First, we imported the series of XPEEM images into the SMART software and exported them as PNG files. Then, we opened the exported PNG images in ImageJ. We then utilized the "Template Matching" feature and obtained the drift-corrected images. These corrected images were then exported and re-imported into the SMART software. To extract the spectra, we selected a small ROI on top of the particle and STO support using the "Define Area of Interest" menu, and created one or multiple ROIs of the desired size. Then, all images were selected in the right panel and the selected area was integrated for each of the ROI. Finally, we saved the file containing the spectra in an ASCII format and opened it in the software Origin for further analysis.

Fit parameters of Rh XP spectra

Table S1 and S2 contain the Rh 3d XPS fit results of Figure 3 such as binding energy (BE), full width-half maxima (FWHM) of Rh5/2 metal and the calculated oxide (I_{Oxide}/I_{Total}) and alloy intensity ratio (I_{Alloy}/I_{Total}) on particle 1 and on the STO support at all analyzed conditions. All binding energies and FWHM are in eV and the intensity ratios are in percent (%).

Table S1: Fitting parameters of Rh on top of Pt particle 1

Treatment	Binding energy (eV)			FWHM (eV)	I_{Oxide}/I_{Total}	I_{Alloy}/I_{Total}
	Rh5/2 metal	Rh5/2 oxides	PtRh alloy	Rh5/2 metal		
RT	306.72	307.72, 308.50	-	0.82	13.8	-
Anneal	306.73	307.54	305.91	0.71	10.7	4.1
$pO_2$1	306.73	307.57	305.95	0.81	12.7	4.2
$pO_2$2	306.73	307.56	305.93	0.72	14.0	4.1
pH_2	306.72	307.80	-	1.06	7.1	4.8

Table S2: Fitting parameters of Rh on the STO support

Treatment	Binding energy (eV)		FWHM (eV)	I_{Oxide}/I_{Total}
	Rh5/2 metal	Rh5/2 oxides	Rh5/2 metal	
RT	306.71	307.66	0.75	10.9
Anneal	306.73	307.63	0.76	8.9
pO1	306.72	307.62	0.90	10.0
pO2	306.72	307.61	0.89	11.9
pH_2	306.71	307.71	0.92	6.7

Pt XPEEM spectra on top of the Pt particle

Figure S3 shows the fitted Pt 4f X-ray photoelectron (XP) spectra on top of the Pt particle 1 at all analyzed conditions. The presence of the Pt doublet peaks 4f7/2 and 4f5/2 at binding energies 71.0 eV and 74.3 eV respectively, resulting from the spin-orbital splitting, are in good agreement with literature.[S7]

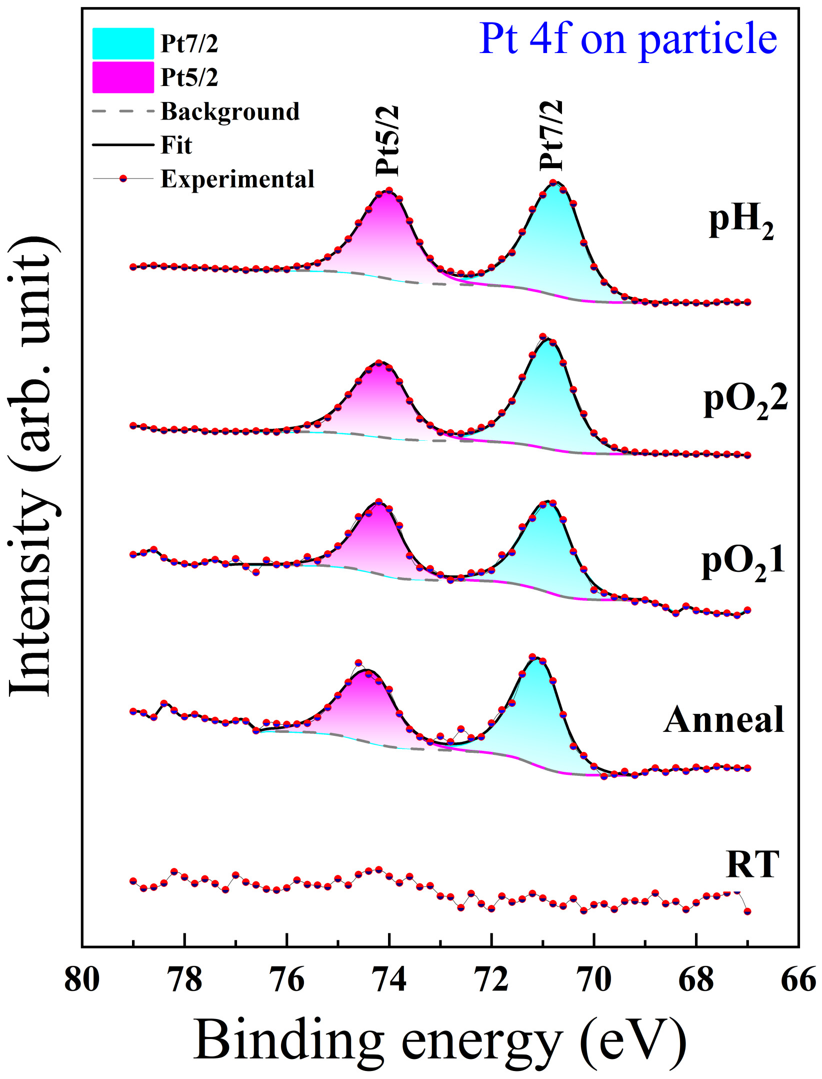

Figure S3: Fitted XP spectra of Pt 4f for each applied condition. Dots indicate the experimental data points. The two broad peaks correspond to Pt 4f7/2 and 4f5/2. The black solid line represents the sum of all fitted contributions and the dashed line is the background.

Scanning Auger microscopy (SAM)

Figure S4 shows the Auger maps of Rh along with Auger survey spectra from the STO support before and after the *in situ* experiment, using an electron acceleration voltage of 20 kV and a beam current of 1 nA. The Rh Auger maps were processed by linear least square fitting utilizing a principal component analysis. See the experimental section for more details. The Rh Auger map before the *in situ* experiment shows higher intensity in the particle region, whereas the intensity decreases after the *in situ* experiment, clearly showing Rh diffusion into the Pt particle. Additionally, the Auger survey spectra clearly show the enhancement of the STO support elements, Sr and Ti after oxidation which indicates a decreased Rh surface coverage due to sintering.

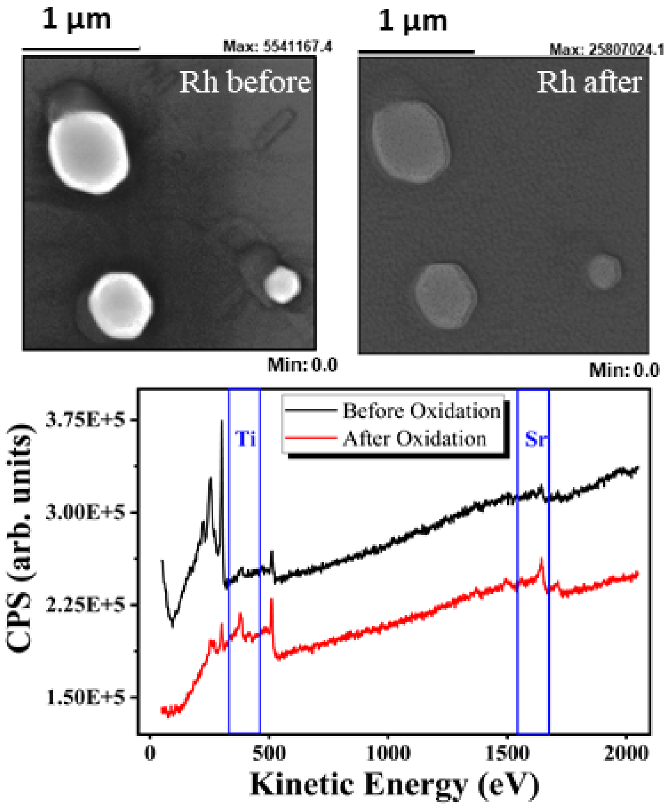

Figure S4: Top: Auger maps of Rh before and after the *in situ* experiment. Bottom: Auger survey spectra before and after the *in situ* experiment from the STO support.

XPEEM spectra of three differently-shaped particles

Figure S5 presents the XP spectra of the Rh 3d5/2 peak for particles 1, 2, and 3, both at room temperature and after the first oxidation. The fitted metallic Rh 3d5/2 peak for all three particles is observed at a binding energy of 306.72 eV. The size and height of the particles, determined via SEM and AFM, are summarized in Table S3, indicating that particle 2 is the smallest, while particle 3 is the largest in diameter and height.

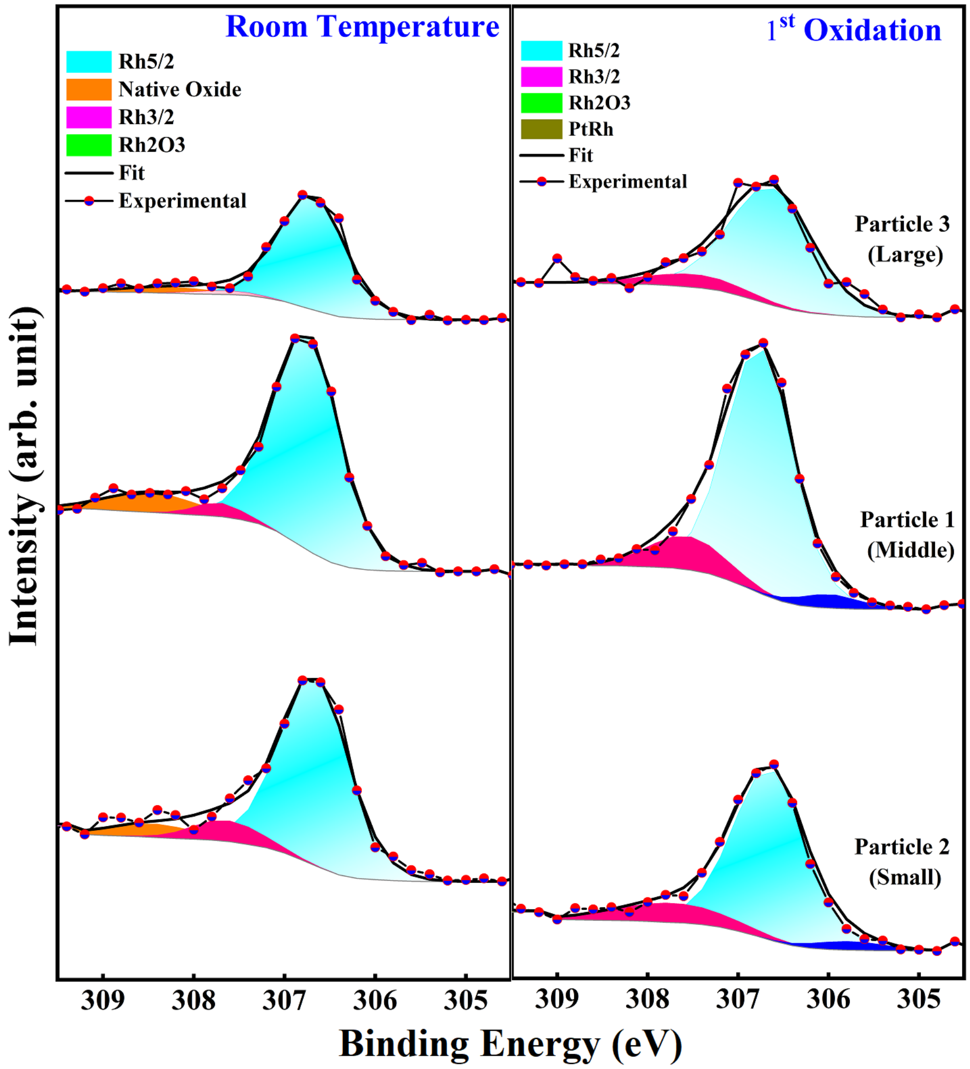

Figure S5: X-ray photoelectron spectra of Rh 3d5/2 on ROIs collected on top of three Pt particles of different size at room temperature and after the first oxidation. Dots indicate experimental data.

Table S3: SEM and AFM results of all three particles.

Particles	Size (nm, SEM)	Height (nm, AFM)
Particle 1 (middle)	739	344.4
Particle 2 (small)	575	201.5
Particle 3 (big)	980	475.6

Particle reconstruction and facet identification

We used the software VESTA with the Pt crystal structure from the ICSD database (Pt-ICSD-Database-entry-76153) to reconstruct the atomic arrangement of the Pt particles.[S8] By combining AFM and EBSD information, we identified altogether six facets, two on particle 1, one on particle 2, and three on particle 3. VESTA is a three-dimensional visualization system for crystallographic studies. Figure S6a, f, and i are 2D top view AFM images, showing particles 1-3 with the in-plane orientations indicated along X and Y, where X and Y are the reference coordinates of the EBSD system. Out-of-plane and in-plane orientations of all the particles, identified by EBSD are summarized in Table S4. Figures S6l, m, and n represent the cross-section view of the AFM images of all the particles. The red dotted arrows in the AFM images of Figure S6 direct in the in-plane direction in which the top surfaces are tilted out-of-plane. First, using VESTA, the particles 1-3 were reconstructed based on the in-plane and out-of-plane orientations from EBSD, see, e.g., Figures S6(b, d, g, j) showing the top view crystal orientations of the particles. For instance, the top view of particle 2 is [111]-oriented (see Figure S6g and Table S4). Figures S6(b, c, d, e, g, h, j, k) show yellow, blue, and green vectors corresponding to the -X, -Y, and Z-orientations of the particles, respectively, along with their corresponding planes, as referenced to the AFM images. The black vector represents the [111] orientation in all images. Further, the in- and out-of-plane angles between the tilted surfaces and the identified Y- and Z-orientations from EBSD were calculated by considering the relative out-of-plane and in-plane rotations necessary to bring a specific facet identified in the AFM image to the top with a parallel alignment to the surface along Z. All calculated out-of-plane and in-plane tilt angles of particle 1, 2, and 3 are listed in Table S4. For example, the Y- and Z-orientations of Particle 2 are [0-33] and [111], respectively. Whereas the top surface has an out-of-plane tilt with respect to the [111] direction of 15° (see Figure S6m), it is also rotated by 15° in-plane with respect to the (-Y)-direction (see Figure S6f). For each of the six facets, we identified the closest-by facet index corresponding to the calculated angles. For Particle 2, the most

probable facet lying at a 15° angle in-plane from (-Y) and out-of-plane from Z is (353). To confirm this, we rotated the particle 15° in-plane around the -Z axis, i.e., counter-clockwise, as the tilted top surface has an in-plane 15° tilt from the (-Y)-orientation. We then rotated it 15° clockwise around the X axis to obtain the top view orientation of the identified facet, followed by a 90° clockwise rotation around the X axis to view the side of the identified facet. Correspondingly, Figure S6h shows the side view of the identified (353) facet, confirming the correct facet identification. Similarly, we identified the indices of all other top facet surfaces of particles 1 and 3 (see Figure S6 and S7) and listed them in Table S4.

Table S4: Out-of-plane and in-plane orientations of all the particles from EBSD. Out-of plane and in-plane tilt angles of their top surface facets along with the obtained facet indices.

Particle #	Out-of-plane orientation (Z), EBSD	In-plane orientation (X, Y), EBSD	Out-of-plane tilt angle (deg)	In-plane tilt angle (along Y or -Y as indicated) (deg)	Identified facets
Particle 1 (middle)	[233]	([-13-2], [-503])<br>([32-1], [-56-2])	26	15 (-Y)	(331)<br>(102)
Particle 2 (small)	[111]	([-211], [0-33])	15	15 (-Y)	(353)
Particle 3 (big)	[536]	([4-1-3], [02-1])	45<br>56<br>62	10 (Y)<br>62 (-Y)<br>30 (-Y)	(131)<br>(-104)<br>(7-75)

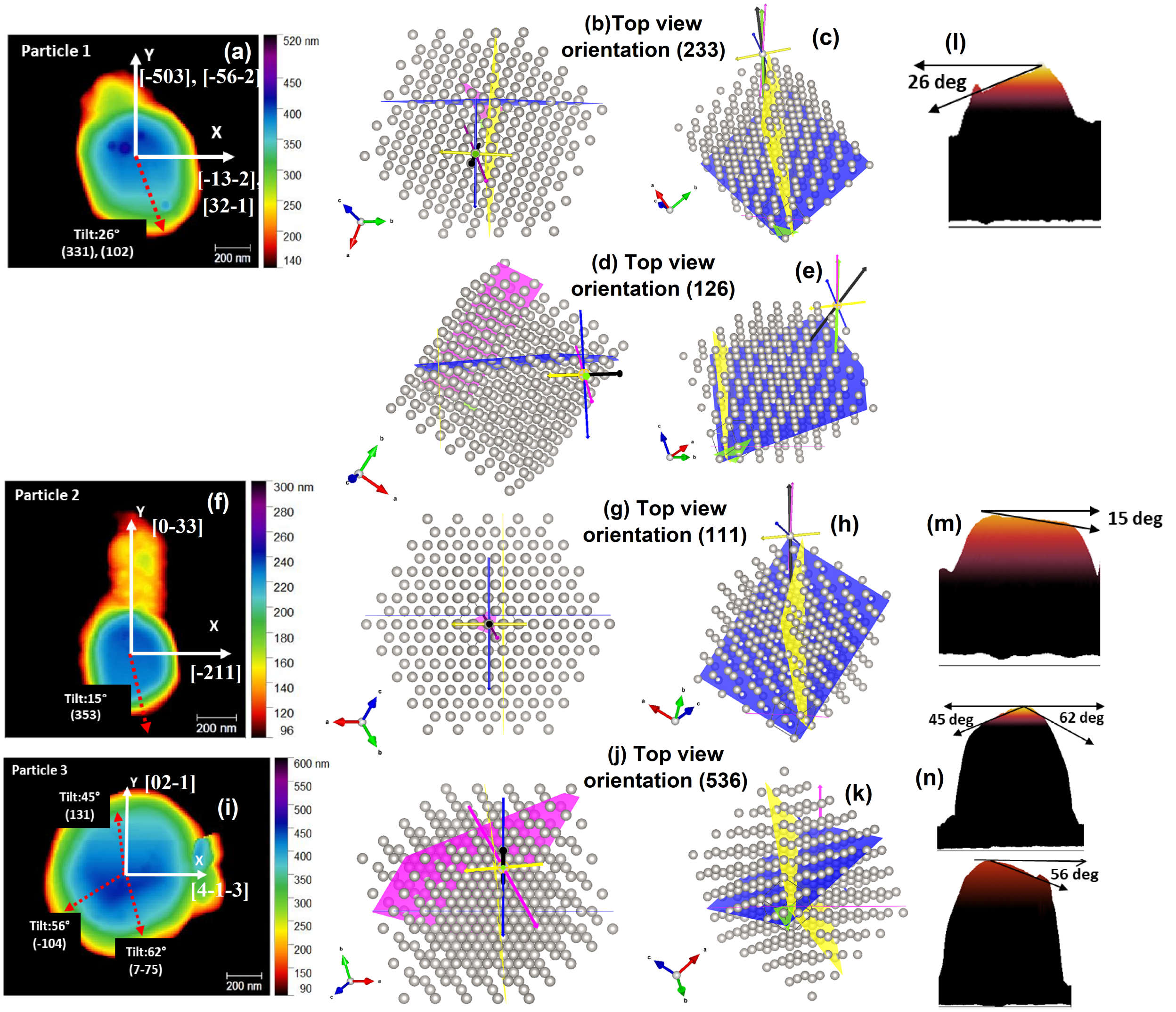

Figure S6: Particle reconstruction in VESTA using the in-plane and out-of-plane orientations from EBSD and AFM of all three particles in line with the 2D AFM height image. (a,f,i) Top view AFM height image along with red arrows pointing in the in-plane direction in which the top surface facet is tilted out-of-plane. The out-of-plane tilt angles are given along with the obtained facet indices for particle 1, 2 and 3. Top view of (b,d) particle 1 for both grains and (g, j) of particle 2 and 3. (c,e,h,k) Side view of the facets identified by EBSD and AFM. Yellow, blue, and green vectors direct along the -X, -Y, and Z orientation of the particles as referenced to the AFM images, respectively, while the yellow, blue, and green planes correspond to the X, Y, and Z planes. The pink plane and vector indicate the identified tilt surface and its corresponding surface normal. (l,m,n) Cross-section view of AFM images of particle 1, 2 and 3 which clearly indicate that the top surface facets of all particles are tilted.

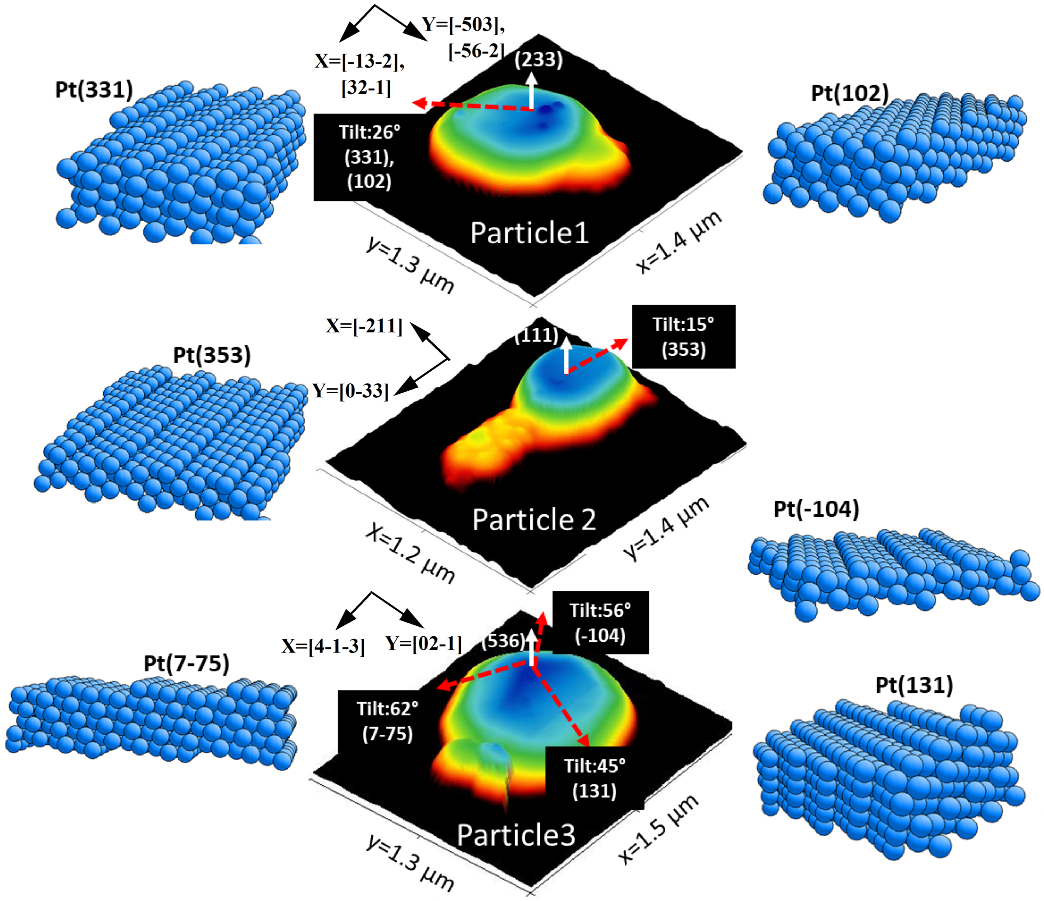

Figure S7: 3D representations of the AFM topography of particles 1, 2 and 3 and atomic distribution of their corresponding surface facets. The tilt angles (see also Figure S6) in the AFM images are given with respect to the substrate normal which is nanoparticle's out of plane crystal orientation (white arrow in 3D AFM images). Black arrows direct along X and Y and are labeled with the corresponding in-plane crystal orientation.

Image registration

Figure S7 display (a) the XPEEM and (b) AFM images registered on the SEM image using the python-based image registration program to localize the ROI on the nanoparticle facets as described in detail in the experimental section.

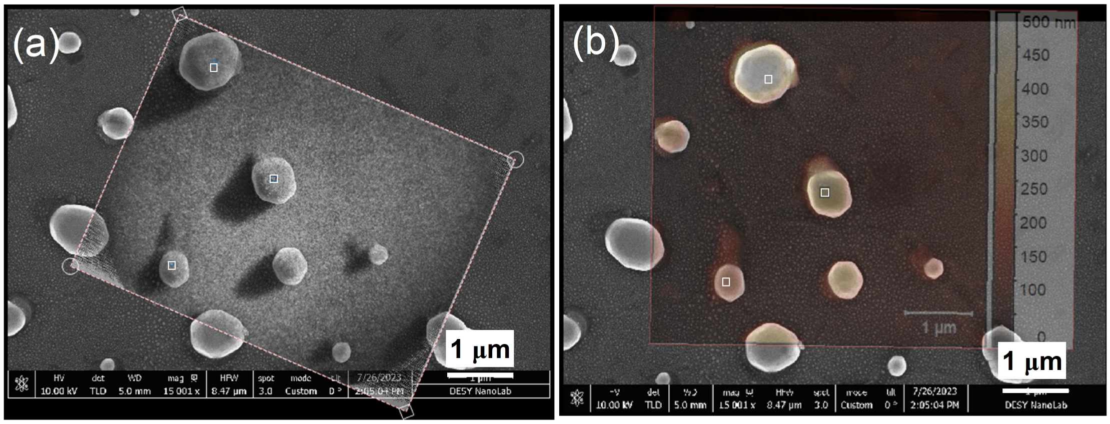

Figure S8: (a) XPEEM and (b) AFM images registered on the SEM image covering all three Pt particles.

References

(S1) Jablonski, A.; Powell, C. NIST Electron Inelastic-Mean-Free-Path Database. *National Institute of Standards and Technology, Gaithersburg* **2010**.

(S2) Shinotsuka, H.; Tanuma, S.; Powell, C. J.; Penn, D. R. Calculations of Electron Inelastic Mean Free Paths. X. Data for 41 Elemental Solids Over the 50 eV to 200 keV Range With the Relativistic Full Penn Algorithm. *Surface and Interface Analysis* **2015**, *47*, 871–888.

(S3) Coey, J. The Crystal Structure of Rh2O3. *Structural Science* **1970**, *26*, 1876–1877.

(S4) Umemoto, K.; Wentzcovitch, R. M. Effect of the d Electrons on Phase Transitions in Transition-Metal Sesquioxides. *Physics and Chemistry of Minerals* **2011**, *38*, 387–395.

(S5) Brundle, C. R.; Crist, B. V. X-ray Photoelectron Spectroscopy: A Perspective on Quantitation Accuracy for Composition Analysis of Homogeneous Materials. *Journal of Vacuum Science & Technology A* **2020**, *38*.

(S6) Schindelin, J.; Arganda-Carreras, I.; Frise, E.; Kaynig, V.; Longair, M.; Pietzsch, T.; Preibisch, S.; Rueden, C.; Saalfeld, S.; Schmid, B.; others Fiji: An Open-Source Platform for Biological-Image Analysis. *Nature Methods* **2012**, *9*, 676–682.

(S7) Budner, B.; Tokarz, W.; Dyjak, S.; Czerwiński, A.; Bartosewicz, B.; Jankiewicz, B. A Novel Approach to Pulsed Laser Deposition of Platinum Catalyst on Carbon Particles for Use in Polymer Electrolyte Membrane Fuel Cells. *Beilstein Journal of Nanotechnology* **2023**, *14*, 190–204.

(S8) Momma, K.; Izumi, F. VESTA 3 for Three-Dimensional Visualization of Crystal, Volumetric and Morphology Data. *Journal of Applied Crystallography* **2011**, *44*, 1272–1276.
